# Comparative Analysis of Nickel–Phosphine Complexes with Cumulated Double Bond Ligands: Structural Insights and Electronic Interactions via ETS-NOCV and QTAIM Approaches

**DOI:** 10.3390/molecules29020324

**Published:** 2024-01-09

**Authors:** Tímea R. Kégl, Tamás Kégl

**Affiliations:** 1 National Laboratory of Renewable Energy, University of Pécs, H-7624 Pécs, Hungary; trkegl@gamma.ttk.pte.hu; 2 Department of General and Inorganic Chemistry and HUN-REN Research Group for Selective Chemical Syntheses, Hungary, University of Pécs, H-7624 Pécs, Hungary

**Keywords:** nickel(0), energy decomposition analysis, QTAIM, allene, ketene, diazomethane

## Abstract

This study presents a comprehensive analysis of nickel–phosphine complexes, specifically Ni(PH_3_)_2_(OCCH_2_), Ni(PH_3_)_2_(H_2_CCO), Ni(PH_3_)_2_(H_2_CCCH_2_), Ni(PH_3_)_2_(NNCH_2_), and Ni(PH_3_)_2_( $\eta^{1}$ -H_2_CNN). Utilizing ETS-NOCV analysis, we explored orbital energy decomposition and the Hirshfeld charges of the ligands, providing insights into the electronic structures and donor–acceptor interactions within these complexes. The interactions in the ketene and allene complexes exhibit similar deformation densities and NOCV orbital shapes to those calculated for Ni(PH_3_)_2_(NNCH_2_), indicating consistent interaction characteristics across these complexes. The total interaction energy for all $\eta^{2}$ complexes is observed to be over 60 kcal/mol, slightly exceeding that of the analogous carbon dioxide complex reported earlier. Furthermore, the study highlights the stronger back-donation as compared to donor interactions across all $\eta^{2}$ complexes. This is further corroborated by Hirshfeld analysis, revealing the charge distribution dynamics within the ligand fragments. The research offers new perspectives on the electron distribution and interaction energies in nickel–phosphine complexes, contributing to a deeper understanding of their catalytic and reactive behaviors.

## 1. Introduction

**Figure 1:**
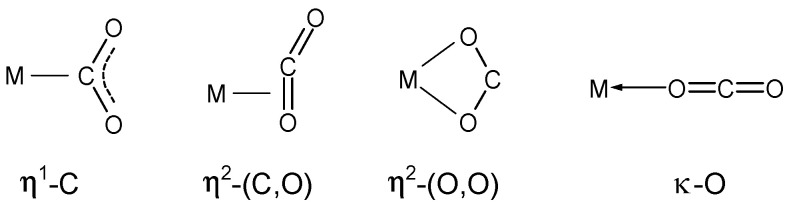
The generally assumed coordination modes of CO_2_ to transition metals.

Reactions of carbon dioxide complexes with transition metals deserve special attention, as CO_2_ can be considered an inexhaustible raw material source and is used as a C1 building block for the production of various organic compounds (formic acid, methanol, and cyclic carbonates) [1,2,3]. Gaining knowledge about the coordination of carbon dioxide to metals (Figure 1) can provide valuable information, which can help develop more efficient catalytic systems [4].

Among the catalytic reactions based on carbon dioxide substrates, hydrogenation is noteworthy [5,6], which results in the formation of formic acid. The reaction was discovered by Farlow and Adkins in 1935, using Raney nickel as a catalyst [7]. The first application in homogeneous catalysis was published by Inoue and colleagues in 1976 [8]. With suitable catalysts, such as the Pd-dppm complex, the reduction can proceed to the formation of methane [9,10]. Other noteworthy homogeneous catalytic reactions based on CO_2_ activation include rhodium-catalyzed hydrosilylation [11,12,13], as well as coupling reactions with alkenes [14,15,16] and alkynes [17].

The diazo group of diazoalkanes can be formally exchanged for the isoelectronic carbon monoxide. The resulting ketenes are versatile, generally highly reactive intermediates in organic syntheses [18,19,20,21,22,23,24]. The carbonylation of diazomethane was first published by Staudinger and Kupfer [25]. For the production of ketene, carbon monoxide was bubbled through a 1% ether solution of diazomethane, and the resulting vapor/gas mixture was heated in a quartz tube above 400 °C (Equation (1)). The exiting gas, when reacted with an ether solution of aniline, yielded acetanilide, which indirectly proved the formation of ketene (Equation (2)).

(1) $${\mathrm{CH}}_{2}N_{2}+\mathrm{CO}\overset{\Delta }{\underset{-N_{2}}{\to }}{\mathrm{CH}}_{2}=C=O$$

(2) $${\mathrm{CH}}_{2}=C=O\overset{{\mathrm{PhNH}}_{2}}{\to }\mathrm{PhNHC}(=O){\mathrm{CH}}_{3}$$

Ketenes are generally produced from the corresponding carboxylic acids or carboxylic acid derivatives. The industrial production of unsubstituted ketene is performed via pyrolysis of acetic acid [26]:

(3) $${\mathrm{CH}}_{3}\mathrm{COOH}\overset{\Delta }{⇌}H_{2}C=C=O+H_{2}O$$

Previously, we studied the carbonylation of diazoalkanes in the presence of various cobalt catalysts as well as the mechanism of the reaction [27,28,29]. Moreover, the mechanism of diazocarbonylation was scrutinized for the nickel–carbonyl- [30] and the iron–phosphine–carbonyl- [31] catalyzed model reactions as well.

In connection with our previous studies, the goal of the present study is to scrutinize the electronic structure of complexes Ni(PH_3_)_2_( $\eta^{2}$ -(O,C)-OCCH_2_) (**1**), Ni(PH_3_)_2_( $\eta^{2}$ -(C,C)-H_2_CCO) (**2**), Ni(PH_3_)_2_( $\eta^{2}$ -(C,C)H_2_CCCH_2_) (**3**), Ni(PH_3_)_2_( $\eta^{2}$ -(N,N)NNCH_2_) (**4**), and complex Ni(PH_3_)_2_( κ -C-H_2_CNN) (**5**), as well as that of the standalone ketene, allene, and diazomethane ligands.

## 2. Computational Details

All the structures were optimized without symmetry constraints with tight convergence criteria using the Gaussian suite of programs [32], with the exchange and correlation functionals developed by Perdew, Burke, and Ernzherhof [33] and denoted as PBEPBE. For all the atoms, the def2-TZVP basis set [34] was employed. Natural bond orbital (NBO) analyses have been performed using the GENNBO 7.0 program [35]. QTAIM (Quantum Theory of Atoms In Molecules) analyses of the wave function [36] were carried out with the AIMAll software (Version 19.10.12) [37]. For the EDA-NOCV (Energy Decomposition Analysis in combination with the Natural Orbitals of Chemical Valence methodology) calculations, the ADF part of the AMS 2023 software was used [38], employing the PBEPBE functional in combination with the triple- ζ STO basis set for all atoms with one set of polarization functions (denoted as TZP) and a small frozen core. The EDA-NOCV method, also known as ETS-NOCV (where ETS stands for extended transition state), is an attractive method for studying metal–ligand interactions in a wide variety of complexes [39,40,41,42,43,44,45,46,47,48]. For the Domain-averaged Fermi hole analyses, the WinFermi program was used [49].

## 3. Results and Discussion

As carbon dioxide can be considered as the ’prototype’ of the other small molecules with a cumulative double bond, some of its properties will be discussed first followed by those of the other standalone ligands. The electronic structures of nickel(0) complexes with carbon dioxide, carbonyl sulfide, and carbon disulfide were reported previously [50,51,52]. The Cartesian geometries as well as internal coordinates of all species occuring in this study are in the Supporting Information.

### 3.1. The Electron Structure of Carbon Dioxide

**Figure 2:**
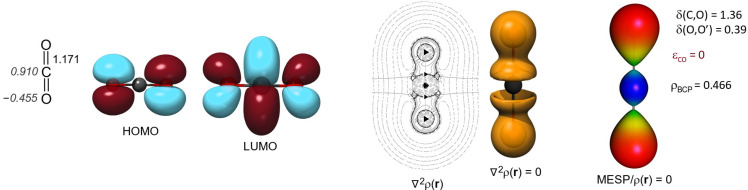
Structure and NPA charges of carbon dioxide calculated at the PBEPBE/def2-TZVP level; HOMO and LUMO orbitals; Laplacian distribution in the plane of the molecule, along with the $\nabla^{2}\rho \left(r\right)=0$ envelope; and the MESP and electron density at the bond critical point, as well as the delocalization indices and bond ellipticity.

The CO_2_ molecule is generally depicted with cumulated double bonds, in which the carbon atom is in an sp hybrid state. In the ground state of carbon dioxide, the $1\pi_{g}$ orbital is the degenerate HOMO, which is an antisymmetric linear combination of the p orbitals centered on oxygen (Figure 2), while the LUMO ( $2\pi_{u}$ ) is more significantly located on the carbon atom. The frontier orbitals provide a fairly good qualitative description of the coordination of CO_2_ to various metals, where the oxygen’s lone pair participates as a donor ligand, while the back-donation interaction is directed towards the carbon atom. However, in stark contrast to the cumulated representation mode (where the symmetry planes of the π orbitals are perpendicular to each other), CO_2_ follows $D_{\infty h}$ symmetry, i.e., it is cylindrically symmetrical [53].

The distribution of electron density can be visually represented by its second derivative with respect to spatial coordinates, i.e., the Laplacian distribution ( $\nabla^{2}\rho \left(r\right)$ ). In Figure 2, the planar representation mode indicates regions with less electron density using dashed lines. Such is the immediate vicinity of the carbon atom, where the molecule shows Lewis acid character. The area enclosed by continuous lines indicates electron density accumulation, suggesting Lewis base character. The figure also shows lines of zero gradient, which separate atomic basins, i.e., the attraction spheres of the atomic nuclei. The surface of the zero Laplacian value also separates areas of electron accumulation and Lewis acid character regions. It is worth mentioning that, although not adjacent to each other, the exchange between the two oxygen atom basins (i.e., the δ (O,O) indicated and is somewhat related to the bond order delocalization index) is surprisingly high: 0.39. Bader and co-workers obtained a value of 0.38 at the HF level and 0.31 at the CISD level for the same delocalization index [54].

**Figure 3:**
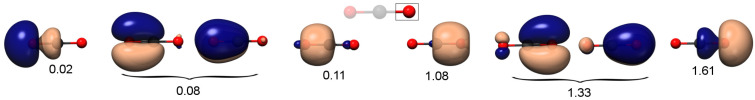
The DAFH eigenvectors of the CO_2_ O fragment, each corresponding to an electron pair, and their associated eigenvalues.

In the next step, using domain-averaged Fermi hole analysis, we examine what the valence electron pairs look like that ultimately result in a cylindrically symmetrical electron density distribution. For our studies, we consider one of the oxygen atoms as the domain (fragment) and depict the eigenvalues of the Fermi hole integrated over it in Figure 3. Each eigenvector corresponds to an electron pair, and the associated eigenvalues indicate the proportion that each pair can be attributed to the given fragment. The σ bond is almost evenly distributed between carbon and oxygen (54%:46%), but interestingly, about 20% of the oxygen’s σ -symmetry lone pair is located on the other (i.e. CO) fragment. The $\pi_{\mathrm{CO}}$ bond is significantly more polarized, being 66.5% attributable to the oxygen fragment. We also find two of them, which are degenerate and, of course, perpendicular to each other.

Inspecting the distribution of electron pairs between fragments, the significant exchange between the basins of the non-adjacent oxygen atoms ( δ = 0.39) becomes more plausible. It is apparent that the $\pi_{\mathrm{CO}}$ orbital, though to a small extent, extends to the second oxygen atom. In addition, the π bonds located on the CO fragment also extend 4% to the O fragment, but the $\pi_{\mathrm{CO}}$ orbital, indeed the $n_{O}$ σ -symmetry lone pair, also appears on the domain we examined at 5.5% and 1%, respectively.

**Figure 4:**
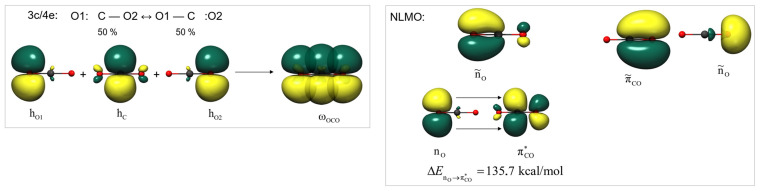
The three-center four-electron π electron system of carbon dioxide (**left**); the natural localized orbitals (NLMOs); and the dominant donor–acceptor NBO interaction in the case of the NLMO functioning as the second π bond.

Observing the eigenvectors, it is noticeable that, contrary to the cumulative representation, there is only one σ -symmetry lone pair on the oxygen atoms, but two completely identical π bonds (which are perpendicular to each other) are found. Therefore, it seems logical to assume that the “disappearing” π -symmetry lone pairs are the sources of the “new” π bonds. To confirm this, we use the natural localized molecular orbitals (NLMOs), which are also suitable for visualizing individual electron pairs. The NLMO can be derived from its parent NBO orbital, whose occupancy of less than two can be supplemented to two via intramolecular donor–acceptor interactions. The 1.62 occupancy of the π -symmetry lone pair centered on the oxygen of CO_2_ is primarily the result of significant electron density transferred to the adjacent CO fragment’s $\pi^{*}$ antibonding orbital, as observed in Figure 4. The resulting NLMO from this transition, characterized by significant interaction energy (135.7 kcal/mol), appears as a highly polarized π orbital, showing similarity to the corresponding DAFH (Domain Averaged Fermi Hole) eigenvector, although the electron pair shape is not entirely identical to that derived purely from the π NBO ( ${\tilde{\pi }}_{\mathrm{CO}}$ ). Similarity can also be found between the NLMO of the σ -symmetry lone pair ( ${ñ}_{O}$ ) and its DAFH eigenvector.

The π electron system of CO_2_ can also be described by the NBO method as a three-center, four-electron bond. A combination of one-center hybrid orbitals based on the $p_{z}$ orbitals found on the two oxygen atoms and the carbon atom can yield two resonance states, which are equivalent to each other and have equal probability.

(4) O1:C−O2↔O1−C:O2(50%:50%)

Both states contain a π -symmetry lone pair on the oxygen and a carbon–oxygen π bond. However, averaging out the two boundary structures, we can also arrive at a 3c/4e bond ( ω -bond), in which the four π electrons are delocalized across the three atoms of CO_2_.

In conclusion, it can be stated that the cylindrically symmetrical structure of CO_2_ can be interpreted using Bader analysis, the NBO method, and DAFH analysis, while also resolving the apparent contradiction in the appearance of the two π bonds assigned to carbon.

### 3.2. Electron Structure of Small Molecules Containing Cumulated Double Bonds

**Figure 5:**
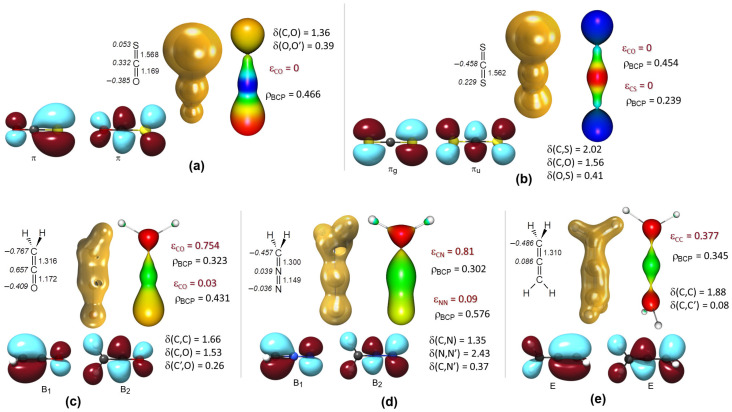
The NPA charge distribution, HOMO (left) and LUMO (right) orbitals, and Laplace distribution with the $\nabla^{2}\rho \left(r\right)=0$ envelope surface; the MESP and electron density at the bond critical point value; as well as the delocalization indices and bond ellipticities of carbonyl sulfide (**a**), carbon disulfide (**b**), ketene (**c**), diazomethane (**d**), and allene (**e**).

We continue our investigations with carbonyl sulfide and carbon disulfide and compare these to ketene, diazomethane, and allene (see Figure 5). Replacing the oxygen atoms in carbon dioxide with sulfur causes significant changes in the electron density distribution of the molecule. For carbonyl sulfide, the molecular symmetry naturally changes from $D_{\infty h}$ to $C_{\infty v}$ . Here, the partial charge of the carbon atom is less positive than in the case of carbon dioxide, but for carbon disulfide, the direction of bond polarity reverses, as the carbon atom’s charge is negative and the sulfur atoms are positive. In the case of ketene, diazomethane, and allene, the methylene carbon carries a partial negative charge, especially pronounced at the terminal carbon atom of ketene.

The C=C and C=N bonds involving the terminal methylene group function as true double bonds, as there is no lone pair available that could be the source of a second double bond. The bond ellipticity is smallest in the case of allene ( $\varepsilon_{\mathrm{CC}}=0.377$ ), followed by ketene ( $\varepsilon_{\mathrm{CC}}=0.754$ ), and then diazomethane ( $\varepsilon_{\mathrm{CN}}=0.810$ ). In the ketene CO and diazomethane CN fragments, the electron density distribution is already very close to cylindrical symmetry. Regarding diazomethane, it is also worth mentioning that according to Ponec and Cooper’s DAFH calculations, there is a polar $\sigma_{\mathrm{CN}}$ and a less polar $\pi_{\mathrm{CN}}$ bond between the CH_2_ and N_2_ fragments [55].

All the examined molecules containing cumulated double bonds show considerable similarity in both HOMO and LUMO orbitals. Except for allene, a rather high delocalization index (0.26–0.41) exists between non-adjacent atoms in the cumulated system, whereas in allene δ (C,C’) = 0.08, meaning that the three-center π electron system characteristic of the other molecules does not form, which can otherwise be characterized as a consequence of an adjacent π → $\pi^{*}$ donor–acceptor interaction u sing the NBO method.

**Figure 6:**
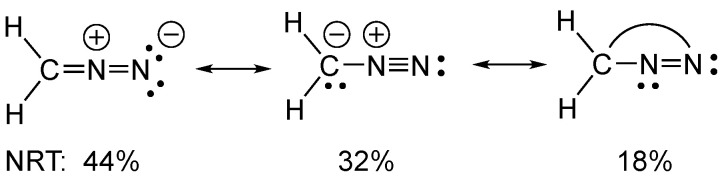
The Natural Resonance Structures of diazomethane and their associated NRT probabilities.

In the case of diazomethane, the probabilities were examined that are assignable to each resonance structure using NRT (natural resonance theory). Although the Lewis resonance structure carrying a positive charge on the internal nitrogen atom is the most probable (44%), surprisingly, the structure indicating a bond between carbon and the terminal nitrogen atom is also assigned a probability of 18% (see Figure 6).

### 3.3. Electron Structure of Metal Complexes Containing Ligands with Cumulated Double Bonds

The ratio of potential to kinetic energy calculated at the bond critical point provides additional information for characterizing a given chemical bond. If 1 <| V(**r**) |/ G(**r**) < 2, it indicates a donor–acceptor-type bond. A value less than one suggests ionic interaction, while |V(**r**) |/ G(**r**) > 2 implies a classical ’shared-shell’ covalent interaction, i.e., when the fragments forming the bond each contribute one electron to the bond.

**Figure 7:**
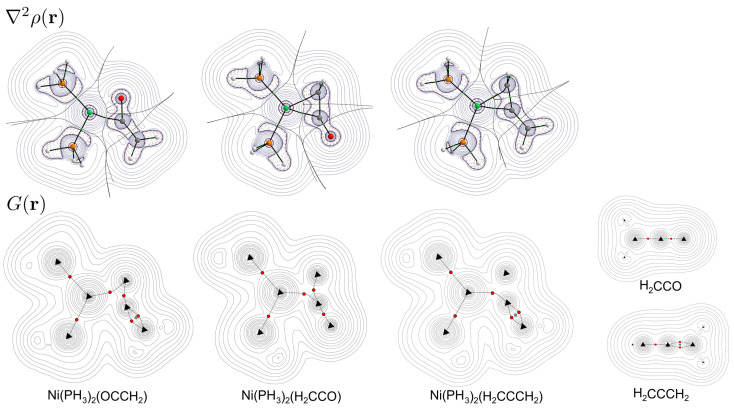
Laplacian (**top**) and kinetic energy density (**bottom**) maps of the Ni(PH_3_)_2_(ketene) and Ni(PH_3_)_2_(allene) complexes. Red dots indicate critical points on the kinetic energy density paths.

The bond paths, Laplacian distribution, and kinetic energy density map of ketene and allene complexes containing the Ni(PH_3_)_2_ fragment can be seen in Figure 7. For ketene (besides the less stable $\eta^{1}$ -O coordination), two types of $\eta^{2}$ coordination modes can be envisioned. Of the two, the $\eta^{2}$ -(C,C) coordination proved to be more stable, with a free energy difference of 5.1 kcal/mol at the PBEPBE/def2-TZVP level. It can be determined that, in terms of electron density distribution, the $\eta^{2}$ -(O,C) complex of the two ketene complexes is more similar to the analogous Ni(PH_3_)_2_(CO_2_) (**6**) carbon dioxide complex, while the $\eta^{2}$ -(C,C) structure (**2**) resembles the Ni(PH_3_)_2_(H_2_CCCH_2_) allene complex (**3**). Similar to the analogous carbon dioxide complex, the absence of a bond path in the $\eta^{2}$ -(O,C) tautomer (**1**) suggests high kinetic energy density in the Ni-O region, which is confirmed by the kinetic energy density path calculated on the G(r) distribution. However, in the $\eta^{2}$ -(C,C) complexes, a bond path forms between the central metal atom and both coordinating carbon atoms, and the kinetic energy density maximum extends from the metal to the middle atom of the ligand.

Among the diazomethane complexes, two proved to be stable; the “side-on” $\eta^{2}$ -(N,N) coordination **4** and the κ -C coordination **5**. The difference in free energy here is 8.4 kcal/mol in favor of the former complex when the two nitrogen atoms are involved in the coordination to the metal center. In our previous studies on nickel–carbonyl [30] and iron–carbonyl–phosphine [31] complexes, no $\eta^{2}$ -(N,N) coordination was predicted; however, κ -N coordination was also found, in contrast with the nickel–phosphine system used in this study.

**Figure 8:**
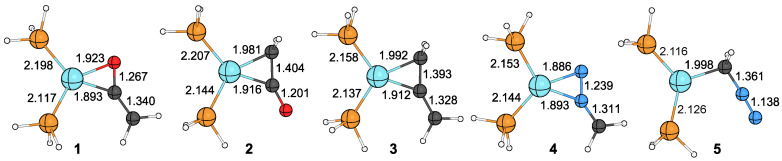
The PBEPBE/def2-TZVP level’s calculated structures of the Ni(PH_3_)_2_(ketene), Ni(PH_3_)_2_(allene), and Ni(PH_3_)_2_(diazomethane) complexes.

The calculated structures of the Ni(PH_3_)_2_ fragment’s ketene, allene, and diazomethane complexes are shown in Figure 8, while their Bader parameters and the Wiberg bond indices (WBIs) of some important bonds are summarized in Table 1. In all cases, the coordination sphere of the nickel center adopts a planar arrangement with no out-of-plane angles for any of the ligands.

The nickel–phosphorus distances are good indicators that the third ligand can be involved in donor–acceptor interactions, transferring electron density to the $\sigma_{\mathrm{NiP}}^{*}$ orbitals. While the $\eta^{2}$ -(N,N) diazomethane and the $\eta^{2}$ -(C,C) allene complexes show no significant difference in this regard, it is interesting to compare the Ni(PH_3_)_2_(ketene) complexes. Switching the coordination type from $\eta^{2}$ -(O,C) to $\eta^{2}$ -(C,C) results in a noticeable elongation in the Ni-P bonds. That might indicate that the higher thermodynamic stability of complex **2** is due to the more favored overlap between the $\pi_{\mathrm{CC}}$ orbitals of the coordinated ketene with the $\sigma_{\mathrm{NiP}}^{*}$ antibonding orbitals.

Based on the electron density and WBI calculated at the bond critical point, the strength of the interaction between nickel and the internal carbon atom in ketene complexes is similar to that in the carbon dioxide complex, while it is weaker in the allene complex. In the $\eta^{2}$ -(N,N) diazomethane complex, there is almost no difference between the two nitrogen atoms and the metal in terms of $\rho_{\mathrm{BCP}}$ , but according to the Wiberg bond indices, the bond between nickel and the terminal nitrogen is stronger. In the $\eta^{1}$ -C complex, the strength of the interaction between the ligand and the metal is significantly weaker, in line with the greater distance between the metal and the coordinating carbon atom compared to the nickel–nitrogen distances in the $\eta^{2}$ -(N,N) complex. Interestingly, despite the less favored ligand coordination, the metal–carbon distance in complex **1** is shorter than in complex **2**.

The ellipticity of the bonds slightly exceeds the ε value calculated for the Ni-C bond in the carbon dioxide complex. In the $\eta^{2}$ -(C,C) complexes, an extreme degree of flattening is observed in the bond between the central atom and the terminal carbon atom. In the $\eta^{2}$ -(N,N) diazomethane complex, there is also a difference in the ellipticity of the interactions between the two nitrogen atoms and nickel, but here the bond involving the terminal nitrogen does not show such an outstanding ε value.

**Figure 9:**
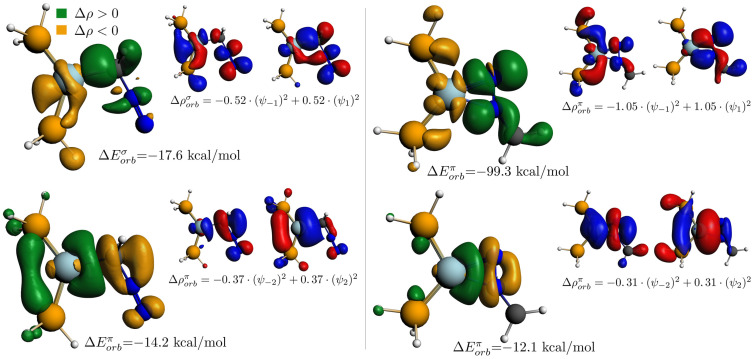
The π -acceptor (**top**) and π -donor (**bottom**) NOCV deformation densities of the Ni(PH_3_)_2_(CH_2_NN) **5** (**left**) and Ni(PH_3_)_2_(NNCH_2_) **4** (**right**) complexes and the corresponding complementary NOCV orbitals.

The dominant donor and back-donation interactions of the Ni(PH_3_)_2_ fragment’s diazomethane complexes are presented in Figure 9. Based on the ETS-NOCV method (Extended Transition State theory combined with the Natural Orbitals of Chemical Valence), in both cases, the back-donation is characterized by greater interaction energy; however, in the $\eta^{1}$ -C complex, this energy value is significantly lower (−17.6 kcal/mol) compared to that calculated in the $\eta^{2}$ -(N,N) complex (−99.3 kcal/mol). This significant difference is well interpretable based on the NOCV eigenvectors. While in the **4** complex, the electron transition largely originates from the nickel $d_{x^{2}-y^{2}}$ orbital and primarily reaches the diazomethane’s π symmetry LUMO, in the **5** complex, the primary source of electron transition is the Ni atom’s $d_{z^{2}}$ orbital, which allows for a much weaker overlap with the LUMO of the H_2_CNN fragment.

The primary source of the donor interaction in both diazomethane complexes is the diazomethane HOMO orbital, while the main component of the NOCV eigenvector, which represents the destination of the electron transition, is the nickel 4*s* orbital. In the case of the ketene and allene complexes, the nature of the interactions, i.e., the deformation densities and the shapes of the NOCV orbitals, do not significantly differ from those calculated for Ni(PH_3_)_2_(H_2_CNN); hence, they are not separately highlighted as figures.

The total interaction energy for all $\eta^{2}$ complexes is very similar, slightly more than 60 kcal/mol (see Table 2). For exception, the carbon dioxide complex was found previously [50], where $\Delta E_{int}$ = 51.9 kcal/mol. The exceptionally high orbital energy value calculated for the $\eta^{2}$ -(N,N) complex (—123 kcal/mol, at least 10 kcal/mol higher than in all other cases) is compensated by the high steric interaction energy (59.6 kcal/mol), which can be described as the sum of the electrostatic and Pauli interactions.

Although not to the same extent as in the **4** complex, but in all $\eta^{2}$ cases, the back-donation is much stronger than the donor interaction. The Hirshfeld analysis of the fragments also confirms this, as the charge on the ligand fragment is least negative in the case of the allene complex **3** (—0.283), exceeding the —0.3 value in other complexes. In contrast, in the $\eta^{1}$ -C diazomethane complex (**5**), there is hardly any charge difference between the two fragments. The minimal negative charge on the diazomethane fragment (—0.038) is consistent with the small difference calculated between the coordination and back-donation interaction energies.

### 3.4. Summary and Conclusions

This research focused on the detailed analysis of nickel–phosphine complexes, specifically targeting Ni(PH_3_)_2_ with various ligands such as ketene, allene, and diazomethane. The investigation employed QTAIM and ETS-NOCV analyses to dissect the orbital energy components and explore the Hirshfeld charges of the ligands, thereby revealing the intricate electronic structures and interactions within these complexes. The results disclosed herein can be summarized as follows.

- Orbital Energy Decomposition and Ligand Charges: The study revealed that the primary source of donor interactions in diazomethane complexes is the diazomethane HOMO orbital, with the nickel 4*s* orbital being integral to electron transitions. The ketene and allene complexes showed interaction patterns consistent with those in the Ni(PH_3_)_2_(H_2_CNN) complex, suggesting similar electronic behaviors.
- Interaction Energy Analysis: The total interaction energy for all $\eta^{2}$ complexes was found to be slightly over 60 kcal/mol. The carbon dioxide complex emerged as an outlier with a slightly lower interaction energy. This finding enriches our understanding of the energy dynamics within these complexes and their potential reactivity profiles.
- Back-donation versus Donor Interactions: A significant observation was the predominance of back-donation over donor interactions in all $\eta^{2}$ cases. This was supported by Hirshfeld analysis, which revealed the nuances of charge distributions within the ligand fragments and between different complexes.
- Charge Distribution and Interaction Strengths: The study noted that in the $\eta^{1}$ -C diazomethane complex, the charge difference between the two fragments was minimal, aligning with the subtle differences in coordination and back-donation interaction energies.

In conclusion, this research provides a comprehensive electronic and structural analysis of nickel–phosphine complexes with various ligands. The findings contribute significantly to the understanding of the electronic properties of these complexes, potentially guiding future research and applications in catalysis and organic synthesis. The consistency of interaction patterns across different ligands and the insights into their energy dynamics offer valuable information for the development of more efficient catalytic systems and the exploration of new synthetic routes in organic chemistry.

## Figures and Tables

**Table 1:** Wiberg bond indices and QTAIM parameters calculated at the bond critical point for Ni-PH_3_ ketene, allene, and diazomethane complexes.

	Bond	ρ (r)	ε	$\frac{V}{G}$	WBI	Bond	ρ (r)	ε	WBI
Ni(PH_3_)_2_(OCCH_2_)	Ni-C1	0.119	0.274	1.518	0.465	C1-O	0.363	0.073	1.496
(**1**)						C1-C2	0.328	0.389	1.784
Ni(PH_3_)_2_(H_2_CCO)	Ni-C2	0.119	0.257	1.474	0.442	C1-C2	0.294	0.222	1.381
(**2**)	Ni-C1	0.103	1.543	1.369	0.332	C2-O	0.423	0.008	1.891
Ni(PH_3_)_2_(H_2_CCCH_2_)	Ni-C2	0.118	0.344	1.474	0.386	C1-C2	0.303	0.225	1.481
(**3**)	Ni-C1	0.101	8.533	1.318	0.353	C2-C3	0.342	0.302	1.946
Ni(PH_3_)_2_(NNCH_2_)	Ni-N2	0.112	0.663	1.226	0.315	N1-N2	0.472	0.063	1.730
(**4**)	Ni-N1	0.116	0.332	1.304	0.525	N2-C	0.323	0.567	1.456
Ni(PH_3_)_2_(H_2_CNN)	Ni-C	0.097	0.043	1.376	0.264	C-N1	0.270	0.452	1.228
(**5**)						N1-N2	0.596	0.025	2.421

**Table 2:** Ziegler–Rauk ETS-NOCV analysis of Ni(PH_3_)_2_(OCCH_2_) (**1**), Ni(PH_3_)_2_(H_2_CCO) (**2**), Ni(PH_3_)_2_(H_2_CCCH_2_) (**3**), Ni(PH_3_)_2_(NNCH_2_) (**4**), and Ni(PH_3_)_2_( $\eta^{1}$ -H2CNN) (**5**) complexes with orbital energy decomposition and Hirshfeld charges of the ligands ( $Q_{H}$ ). The NOCV eigenvalues are given in parentheses. The ratio of dominant back-donation interaction is given in square brackets, and energy values are in kcal/mol.

Complex	$\Delta E_{\mathrm{str}}$ *	$\Delta E_{\mathrm{orb}}$	$\Delta E_{\mathrm{int}}$	$\Delta E_{d}$	$\Delta E_{\mathrm{bd}}$	$Q_{H}$
Ni(PH_3_)_2_(OCCH_2_)	50.3	−113.1	−62.8	−11.1 (0.31)	−90.7 [%] (0.99)	−0.362
Ni(PH_3_)_2_(H_2_CCO)	41.1	−105.2	−64.2	−15.1 (0.40)	−67.2 [%] (0.80)	−0.320
Ni(PH_3_)_2_(H_2_CCCH_2_)	33.2	−96.7	−63.5	−15.1 (0.37)	−67.8 [%] (0.82)	−0.283
Ni(PH_3_)_2_(NNCH_2_)	59.6	−123.0	−63.4	−12.1 (0.31)	−99.3 [77%] (1.05)	−0.365
Ni(PH_3_)_2_(H_2_CNN)	12.2	−39.2	−27.1	−14.2 (0.37)	−17.6 [58%] (0.52)	−0.038

* Total steric interaction: $\Delta E_{str}=\Delta E_{electrostatic}+\Delta E_{Pauli}$ .

## Data Availability

Data are contained within the article.

## Supplementary Materials

The following supporting information containing the internal energies and Cartesian coordinates of all species occuring in this study can be downloaded at: www.mdpi.com/xxx/s1.
