# Supplementary material for: Comparative Analysis of Nickel–Phosphine Complexes with Cumulated Double Bond Ligands: Structural Insights and Electronic Interactions via ETS-NOCV and QTAIM Approaches
Source: Molecules. 2024 Jan 9;29(2):0. doi: 10.3390/molecules29020324 (PMC11154572; doi:10.3390/molecules29020324)
Supplement: Supplementary file 1 [file molecules-29-00324-s001.zip › molecules-2786804-supplementary.pdf]

Supporting Information to Manuscript entitled ”Comparative Analysis of Nickel-Phosphine Complexes with Cumulated Double Bond Ligands: Structural Insights and Electronic Interactions via ETS-NOCV and QTAIM Approachess”

Tímea R. Kégl, Tamás Kégl

Internal energies (in a.u.) and Cartesian coordinates (in Å) of the compounds occuring in this study

$Ni(PH_3)_2$

E=-2194.13545

Ni	0.00000	0.00000	0.00000
P	0.00000	0.00000	2.10330
P	0.00000	-0.00000	-2.10330
H	0.00000	1.22290	2.84407
H	1.05906	-0.61145	-2.84407
H	1.05906	-0.61145	2.84407
H	-1.05906	-0.61145	2.84407
H	-1.05906	-0.61145	-2.84407
H	0.00000	1.22290	-2.84407

$Ni(PH_3)_2(\eta^2\text{-(C,C)}-H_2CCCH_2)$

E=-2310.73078

Ni	-0.10265	-0.09746	-0.00003
P	-2.24062	-0.39386	-0.00025
P	0.38176	1.98415	0.00020
H	-0.05668	2.84909	-1.05032
H	1.73403	2.44637	-0.00463
H	-0.04748	2.84435	1.05839
H	-2.82603	-1.15366	-1.05890
H	-3.25911	0.61211	0.00475
H	-2.82611	-1.16423	1.05067
C	1.59316	-0.98034	0.00001
C	0.60250	-1.95996	0.00108
C	2.87983	-0.64977	-0.00089
H	0.35281	-2.49483	0.92483
H	0.35248	-2.49638	-0.92169
H	3.66198	-1.41698	-0.00060
H	3.21832	0.38904	-0.00197

$Ni(PH_3)_2(\kappa\text{-C}-H_2CNN)$

E=-2342.79779

Ni	0.318572	-0.041284	0.000934
P	2.386439	-0.491801	-0.007400
P	-0.492556	1.924006	0.002074
H	3.017391	-1.778931	-0.140370
H	-0.232103	2.875411	-1.034866
H	3.179336	-0.125240	1.127766
H	3.259323	0.126858	-0.959890
H	-1.918083	2.142327	-0.020785
H	-0.269216	2.852339	1.068203
N	-2.165192	-1.122435	-0.001806
N	-3.190908	-0.629137	-0.014920
C	-0.899848	-1.624682	0.013047
H	-0.739582	-2.185678	0.945479
H	-0.733536	-2.225118	-0.893011

$Ni(PH_3)_2(\eta^2\text{-(N,N)}-NNCH_2)$

E=-2342.81579

Ni	-0.05725	-0.08328	-0.00584
P	-2.07910	-0.85629	0.00546
P	-0.11225	2.07135	-0.00294
H	-2.32809	-2.14081	-0.56675
H	-1.31068	2.81907	-0.23253
H	-3.19295	-0.18144	-0.58881
H	-2.71209	-1.10845	1.26397
H	0.29787	2.80081	1.16022
H	0.70163	2.80527	-0.92567
N	0.88827	-1.71338	-0.02369
N	1.70464	-0.77399	0.00284
C	2.98148	-0.46529	0.01899
H	3.25971	0.58327	0.04098
H	3.71847	-1.26832	0.00641

$Ni(PH_3)_2(\eta^2\text{-(O,C)}-OCCH_2)$

E=-2346.66752

Ni	0.13242	-0.08782	-0.01299
P	2.30879	-0.39820	-0.03038
P	-0.35038	1.97301	0.04478
H	2.88554	-1.48495	-0.76033
H	2.97628	-0.67254	1.20531
H	3.23285	0.59758	-0.47909
H	-1.35360	2.46780	-0.84599
H	0.59501	3.02865	-0.16368
H	-0.92168	2.49759	1.24664
C	-1.59411	-0.86358	-0.00458
C	-2.90245	-0.57272	0.01654
O	-0.74147	-1.80078	-0.03397
H	-3.64091	-1.37807	0.01015
H	-3.25017	0.45740	0.03630

$Ni(PH_3)_2(\eta^2\text{-(C,C)}-H_2CCO)$

E=-2346.67262

Ni	0.11167	0.10177	0.01274
P	2.33082	0.24111	0.02488
P	-0.58904	-1.93791	0.02571
H	-1.99167	-2.18304	-0.10790
H	-0.38981	-2.76581	1.17847
H	-0.15736	-2.91816	-0.92668
H	2.93367	1.04259	-0.99658
H	3.33817	-0.78569	-0.00867
H	2.91192	0.95219	1.12292
C	-1.54412	1.04352	0.02059
C	-0.49366	1.97893	0.01243

O	-2.70979	0.74933	0.03081
H	-0.28240	2.52573	0.93851
H	-0.29079	2.51688	-0.92071

$H_2C{=}C{=}CH_2$

E=-116.53937

C	0.00000	0.00000	0.00000
C	0.00000	0.00000	1.30816
C	0.00000	0.00000	-1.30816
H	0.00000	0.93312	1.87672
H	0.00000	-0.93312	1.87672
H	0.93312	0.00000	-1.87672
H	-0.93312	0.00000	-1.87672

CO_2

E=-188.47924

C	0.00000	0.00000	0.00000
O	0.00000	0.00000	1.17121
O	0.00000	0.00000	-1.17121

$NNCH_2$

E=-148.63489

N	0.00000	0.00000	0.15373
N	0.00000	0.00000	1.29855
C	0.00000	0.00000	-1.14330
H	0.00000	0.95785	-1.65308
H	0.00000	-0.95785	-1.65308

$OCCH_2$

E=-152.49015

C	0.00000	0.00000	-1.21368
H	0.00000	0.94395	-1.75323
H	0.00000	-0.94395	-1.75323
C	0.00000	0.00000	0.10157
O	0.00000	0.00000	1.27239
